# Failure by congestion of pedicled and free flaps for reconstruction of lower limbs after trauma: the role of negative-pressure wound therapy

**DOI:** 10.1007/s10195-013-0236-0

**Published:** 2013-03-31

**Authors:** L. Vaienti, R. Gazzola, E. Benanti, F. Leone, A. Marchesi, P. C. Parodi, M. Riccio

**Affiliations:** 1 Plastic Surgery Department, IRCCS Policlinico San Donato, Università degli Studi di Milano, Piazza Malan, 20097 San Donato Milanese, Milan Italy; 2 Department of Microsurgery Plastic reconstructive and hand surgery, Azienda Ospedaliero-Universitaria Ospedali Riuniti di Ancona Umberto I, 60126 Ancona, Italy; 3 Plastic Surgery Department, Azienda ospedaliera “Santa Maria della Misericordia”, Università di Udine, Gemona del Friuli, Italy

**Keywords:** Flap, Lower limb, Edema, Congestion, Negative pressure

## Abstract

Lower limb reconstruction with pedicled or free flaps can be commonly compromised by venous insufficiency. This complication often leads to partial/complete flap necrosis and increases the risk of superinfection. Negative-pressure wound therapy (NPWT) is known to increase local blood flow, decrease edema, promote tissue granulation, and reduce the likelihood of soft tissue infection. This study aims to evaluate the effectiveness of NPWT in the treatment of congested pedicled and free flaps of the lower limb after reconstructions in lower limb traumas. A retrospective analysis was performed on four congested (pedicled and free) flaps on the lower limbs. NPWT was applied in all cases after partial flap debridement. NPWT was able to improve and resolve tissue edema and venous insufficiency, avoid further flap necrosis, and promote granulation. On NPWT removal, a split-thickness skin graft was applied on the wound, achieving complete and uneventful healing. NPWT is a useful instrument in managing flaps affected by venous insufficiency in lower limb reconstruction, although larger studies are necessary to better define the effectiveness and indications of NPWT in this setting.

## Introduction

Reconstruction of lower limbs after trauma is a challenging problem for the reconstructive surgeon, in terms of both surgical treatment and postoperative healing.

In fact, local vascularization is often compromised and wounds are commonly contaminated or infected. Venous flap insufficiency, edema, hematomas, hemorrhage, and infections can therefore complicate the reconstructive plan [1] by causing partial or total flap necrosis.

Lorenzo et al. [2] showed that venous insufficiency was more frequent than arterial insufficiency: in a total of 404 free tissue transfers in the lower extremity, 26 flaps suffered from venous insufficiency and 21 arterial. This result was confirmed by other authors also in case of pedicled perforator flaps [3].

Moreover, postoperative edema after flap reconstruction of lower limbs is a common complication which affects most patients affected by extensive traumas. Nevertheless, in the most severe cases, postoperative reactive edema and inflammation can protract the healing course [4]. Negative-pressure wound therapy (NPWT) is known to increase local blood flow and decrease tissue edema. In exposed fractures, negative-pressure dressings have been demonstrated both to encourage tissue granulation [5] and to reduce the likelihood of soft tissue infection [6].

The aim of this work is to study the role of NPWT in the healing process of vascularized pedicled and free flaps in traumatic lower limbs.

We therefore studied NPWT applied in congested and edematous pedicled/free flaps of the leg, raised to reconstruct wide and traumatic soft tissue losses.

## Materials and methods

We retrospectively analyzed four patients affected by persistent edema and venous congestion after reconstruction with pedicled and free flaps. The patients were admitted for extensive wounds with bone exposure from October 2010 to December 2011. Patients affected by postoperative tissue edema which resolved spontaneously were not included in the current study.

Continuous NPWT was applied with foam dressings at 120 mmHg. The following parameters were recorded: wound localization, etiology of complication affecting flap viability, surgical debridements performed after the first procedure, date of NPWT application, NPWT duration, and time required for complete healing.

The study was performed in accordance with the ethical standards of the 1964 Declaration of Helsinki as revised in 2000.

## Results

Four patients were included in the study, two females and two males, affected by extensive soft tissue damage after traffic accidents, involving foot (two cases) and leg (two cases) (Table 1). In one case a free flap was harvested for reconstruction, while in the other cases a pedicled flap was performed. Venous insufficiency was observed on average 3.5 days from surgery. In all cases but one, partial flap debridement was necessary before application of NPWT. The mean duration of NPWT was 17.75 days. The dressing was renewed every 4 days.

**Table 1:** Summary of patient data

No.	Sex	Wound location	Surgery	Complication	Days	Flap debridement	NPWT application	NPWT duration (days)	Further treatment	Days required for complete healing after NPWT application
1	M	Calcanear—right foot	Latissimus dorsi free flap + partial-thickness skin graft	Venous insufficiency	2nd postop.	Yes	25th postop.	14	Partial-thickness skin graft	10
2	F	Calcanear—left foot	Reverse fasciocutaneous sural flap?	Venous insufficiency	4th postop.	Yes	14th postop.	15	Partial-thickness skin graft	11
3	F	Distal third left leg	Reverse fasciocutaneous sural flap	Venous insufficiency	3rd postop.	No	9th postop.	22	Partial-thickness skin graft	9
4	M	Middle third left leg	Hemisoleus flap + partial-thickness skin graft	Venous insufficiency	5th postop.	Yes	13th postop.	20	Partial-thickness skin graft	9

**Fig. 1:**
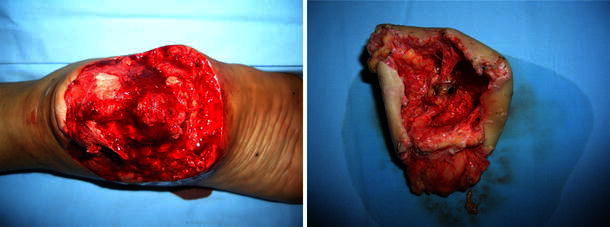
A 27-year-old male affected by extensive damage of soft tissue with calcaneal exposure after a motor-vehicle accident. After serial debridements, with negative cultures, a latissimus dorsi free flap was harvested and applied to reconstruct the affected area. The muscle flap was then covered with a partial-thickness graft with 1:3 meshing

**Fig. 2:**
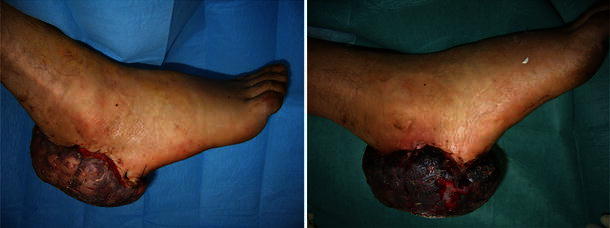
Two days after flap harvesting, venous congestion and flap edema were observed, causing marked flap hypoxic ischemia and partial flap necrosis

**Fig. 3:**
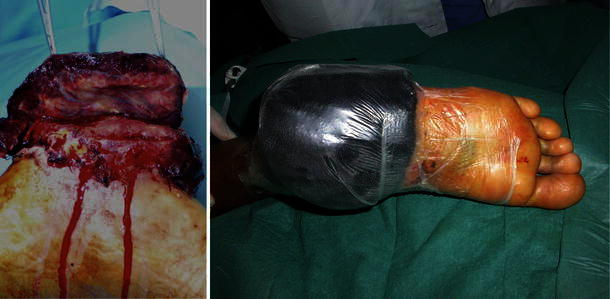
Twenty-five days postoperatively partial debridement of the flap was performed, and NPWT was applied for 2 weeks

**Fig. 4:**
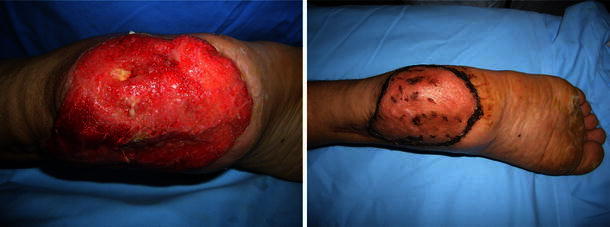
After NPWT removal, the wound showed viable granulation tissue, and a partial-thickness skin graft was employed for wound treatment; complete healing occurred 8 days after NPWT removal

After NPWT removal, viable granulation tissue was observed and the wound could be treated with a partial-thickness skin graft in all cases. Complete healing occurred in 9.75 days after NPWT removal, and the overall reconstructive process lasted 42.75 days on average (Figs. 1–4).

## Discussion

Trauma of the lower limb is often combined with extensive soft tissue damage and impairment of local vascularization. In this setting, flap failure by venous insufficiency and congestion is more common than arterial insufficiency [1].

Venous insufficiency and flap congestion can result from different causes: hematoma and edema inducing compression of the vascular system, twist of the pedicle, and thrombosis can lead to venous insufficiency [7]. In venous insufficiency, the slowing of capillary flow could lead to microthrombosis and finally to arterial insufficiency.

Failure by venous congestion and edema of flaps of the lower limb varies depending on the primary trauma, the systemic condition of the patient, and the surgical technique.

Few procedures to prevent flap congestion have been described in the literature, mainly consisting of surgical re-exploration and application of leeches or compressive stockings [4, 8, 9].

Winterton, in his series of 2,569 flaps, performed flap re-exploration in 13 % of cases. Among these patients, the overall success rate was 95.3 % [8]. Nevertheless, re-exploration is not directly comparable to NPWT. In fact, the former is indicated in case of arterial or venous insufficiency by anastomotic damage in free flaps. Conversely, NPWT should be applied in those cases where flap insufficiency is not directly related to anastomotic damage or in nonmicrovascular flaps.

Lee et al. [10] demonstrated in an animal model the active role of leeches in minimizing flap necrosis after venous congestion. In this case the data concerning the success rate of leech therapy are not clear. NPWT has several advantages in parallel with these techniques. In fact, it directly acts on fluid drainage and reduces the risk of soft tissue infection, promoting bacterial clearance [6], avoiding the risk of further surgical re-explorations and those of leech therapy [11] (e.g., infections with *Aeromonas hydrophila*, bleeding, anemia, allergic reactions, thrombotic microangiopathy, and acute renal failure).

On the other hand, NPWT has been demonstrated to improve vascularization and to induce angiogenesis. Literature suggests that negative pressure increases blood flow and modifies the ultrastructure of capillaries and endotheliocytes, finally promoting the formation of small vessels [12].

Moreover, NPWT encourages take and survival of skin grafts, often employed along with flaps in lower limb reconstruction [13].

Use of NPWT on chronic and traumatic wounds has been described [14]. In traumatic wounds, use of NPWT on compromised flaps is recommended with significant caution [15]. NPWT could be able to increase flap viability, although it could cause compression over the vascular pedicle.

In our study, NPWT was applied on four flaps affected by venous insufficiency. On NPWT removal, the residual flap displayed no necrotic tissue, as well as a marked improvement of edema. No infection was found on NPWT removal.

Disadvantages of NPWT mainly include the immediate cost of the device and rare complications such as toxic shock syndrome, as described by Gwan-Nulla [16], and the potential stimulating action on Gram-negative bacteria [17]. Moreover, the patient with NPWT requires strict follow-up, and accurate fluid balance should be performed to avoid excessive depletion [18].

We believe that NPWT is an essential instrument to manage flaps affected by venous congestion after lower limb reconstruction, although larger studies are necessary to better define the effectiveness and indications of NPWT in this setting.
